# Prevalence and Predictors of Osteoporosis and Osteopenia in Postmenopausal Women of Punjab, India

**DOI:** 10.3390/ijerph19052999

**Published:** 2022-03-04

**Authors:** Rubanpal Khinda, Srishti Valecha, Nitin Kumar, J. P. S. Walia, Kuldeep Singh, Sudhir Sethi, Avtar Singh, Monica Singh, Puneetpal Singh, Sarabjit Mastana

**Affiliations:** 1 Department of Human Genetics, Punjabi University, Patiala 147002, Punjab, India; ruban_rs19@pbi.ac.in (R.K.); srishti_rs19@pbi.ac.in (S.V.); nitin_rs19@pbi.ac.in (N.K.); singhmonica2017@gmail.com (M.S.); 2 Department of Orthopedics, Aggarsain Charitable Hospital, Patiala 147002, Punjab, India; jpsw0124@gmail.com; 3 Department of Orthopedics, Government Medical College and Hospital, Patiala 147002, Punjab, India; kuld49439@gmail.com; 4 Department of Orthopedics, Mata Kaushalya Hospital, Patiala 147002, Punjab, India; sethisudhir196@gmail.com; 5 Department of Orthopedics, Amandeep Hospital, Amritsar 143001, Punjab, India; asgh1962@gmail.com; 6 Human Genomics Lab, School of Sport, Exercise and Health Sciences, Loughborough University, Loughborough LE11 3TU, UK

**Keywords:** prevalence of osteoporosis, osteopenia, independent predictors, postmenopausal women

## Abstract

The prevalence and predictors of osteoporosis and osteopenia remain to be examined in the postmenopausal women of Punjab, India. The present cross-sectional study screened 1628 postmenopausal women during September 2019 to March 2020. Osteoporosis and osteopenia were confirmed on the basis of T-scores using dual energy X-ray absorptiometry (DXA) at the hip (femoral neck) and lumbar spine regions (L1–L4 vertebrae). The prevalence of osteoporosis and osteopenia was observed to be 30.50% and 44.20%, respectively, in postmenopausal women of Punjab. In univariable and multivariable regression analysis, variables independently influencing the risk of osteoporosis and osteopenia were: higher systolic blood pressure (95%CI: 1.22–3.11 & 1.08–2.49), triglyceride levels (95%CI: 1.21–3.10 & 1.42–2.51), poor sleep quality (95%CI: 1.91–2.47 & 1.76–3.47) and C-reactive protein levels (95%CI: 2.18–3.56 & 1.03–2.18). Years since menopause >10 years was observed to be an independent predictor for the risk of osteopenia but not for osteoporosis. Higher body mass index (>30 kg·m^−2^) was observed to be a significant protective factor against the risk of osteoporosis (95%CI: 0.26–0.68) and osteopenia (95%CI: 0.19–0.52). The higher prevalence rates of osteoporosis and osteopenia in postmenopausal women of Punjab are alarming, which solicits awareness and earlier testing of those women who are approaching menopause.

## 1. Introduction

Osteoporosis is a systemic disease whereby complex, composite and complicated molecular pathways interact to reduce the bone mass and its strength by triggering micro-architectural degradation of bone [1]. Decreased bone mineral density (BMD) is a major consequence that is associated with feeble, frail and fractured bones [2]. Osteoporosis and its worst outcomes such as fractures and chronic pain are common in both genders, however, women are more vulnerable, accounting for 70–80 percent of all traumas including hip, spine and wrist fractures [3]. This prevalence increases further in postmenopausal women because of reduced estrogen levels resulting in accelerated loss of bone mass [4].

Several reports have revealed many factors which are associated with osteoporosis and may increase its risk [1,2,3,4]. These factors are female gender, advancing age after menopause, low body mass index (BMI), family history, poor diet, sedentary lifestyle, smoking, alcohol consumption and affiliated comorbidities [1,2,3,4]. The magnitude and impact of these risk factors, individually or in concert, varies due to different geographies [5]. For instance, chances of osteoporosis and its associated fractures are more prevalent in Scandinavian populations than people of Africa and South America, due to lesser exposure of vitamin D in Scandinavia than countries having more annual sunrays and sunshine [6]. Moreover, peak bone mass attainment also differs due to variable nutritional intake in developing and developed countries [2].

According to statistics given by the World Health Organization (WHO), 30 percent of postmenopausal women suffer from osteoporosis [4]. It has been reported that 61 million people in India have osteoporosis and, out of these, 80 percent are women [7]. The peak incidence of osteoporosis in India occurs 10–20 years earlier than in Western countries, which impinges harshly on the health and economic resources [8,9]. Prevalence statistics of postmenopausal osteoporosis and knowledge regarding its independent predictors are lacking, especially in India, where every third woman and every eighth man is suffering from it [7]. Investigation of common risk factors for calculating the independent risk predictors for osteoporosis is an important strategy, which can be useful in formulating an effective approach for managing osteoporosis and its imperative consequences. There is no compelling report on the prevalence and predictors for osteoporosis or osteopenia in the population of Punjab, India, which has been analysed in the present study.

## 2. Subject and Methods

### 2.1. Study Participants

**Figure 1:**
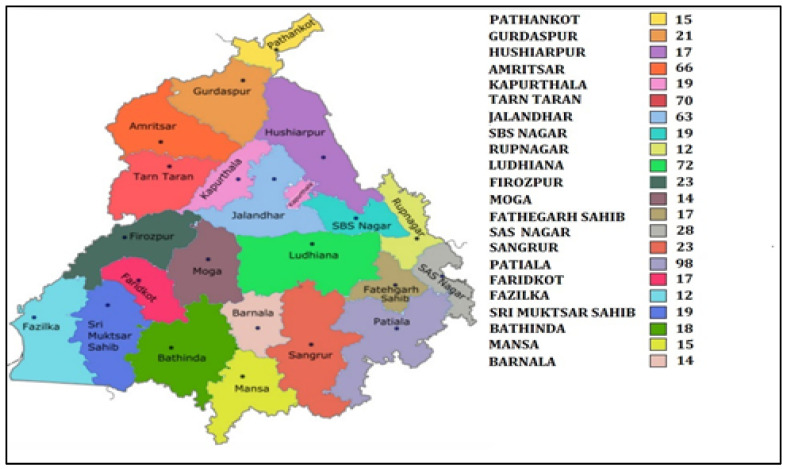
Map of Punjab showing district wise sample collection.

**Figure 2:**
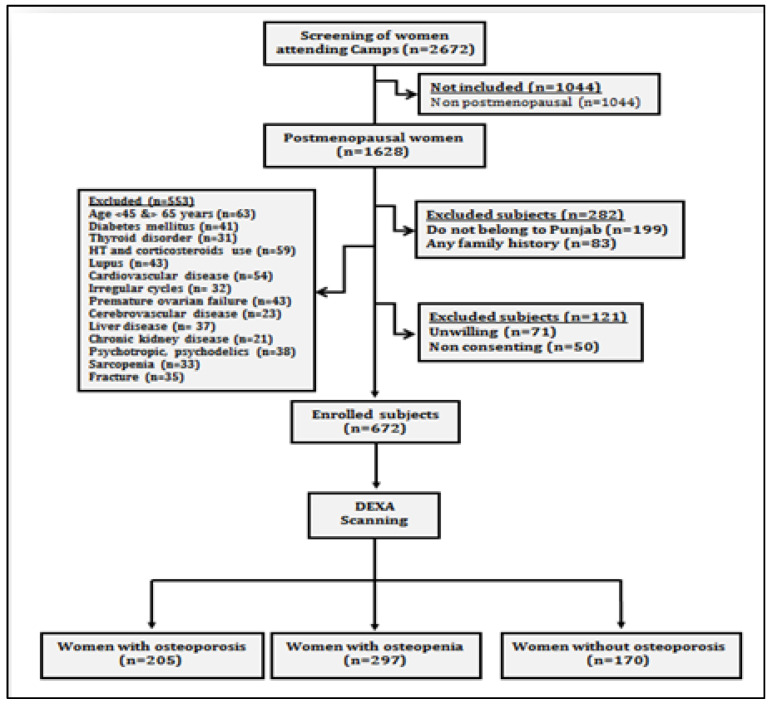
Study flow chart showing data collection protocol.

The present prospective cross-sectional study was carried out on postmenopausal women, who attended general health check-up camps from September 2019 to March 2020. These camps were organized as community service programmes by various hospitals of Punjab (Figure 1). After preliminary screening of 2672 women, 1628 women ranging in age between 50 and 80 years who had confirmed menopause (one or more complete years of cessation of the menstrual cycle) were enrolled for the study. All those women who were non-consenting, having other musculoskeletal disorders, cardiovascular disorders, cerebrovascular pathology, sarcopenia, diabetes, liver disorders, family history of osteoporotic fractures, thyroid dysfunction, all lupus, chronic kidney disease, taking hormone therapy or any medication affecting blood pressure or lipoprotein metabolism, taking psychotropics, psychoactives, psychedelics or multivitamins/antioxidants were excluded (Figure 2). Finally, 672 women were recommended for bone mineral density (BMD) testing. These women were tested with dual energy X-ray absorptiometry (DXA) at the femoral neck (hip) and lumbar region (L1–L4 vertebrae). On the basis of T-scores calculated according to WHO guidelines [10], the subjects were categorized as women with osteoporosis (*n* = 205), women with osteopenia (*n* = 297) and women with normal bone mass (*n* = 170). All the postmenopausal women gave their written consent before participation in the study. The study protocol was approved by the Institutional Ethical Review Committee of Punjabi University, Patiala.

### 2.2. Bone Mineral Density Measurement

BMD of the women in supine position was measured with a Hologic QDR 4500 system (Hologic Inc., Waltham, MA, USA) using DXA. On the basis of T-scores obtained according to WHO guidelines [10], women were characterized as osteoporotic when the T-score was equal to or less than −2.5 standard deviation (SD), osteopenic when the T-score was observed between −1 to −2.5 SD and normal (without bone loss) when the T-score was −1 SD from the optimal peak bone density of healthy young adults of the same sex. The DXA system was calibrated every day before using it with the Phantoms supplied by the manufacturer. The coefficient of variation was observed to be less than 4 percent for the measurements of BMD at the hip and spine.

### 2.3. Risk Variables

Age of the subject and years since menopause (YSM) were recorded from the medical profiles of the patients or by conducting a personal interview. Body mass index (BMI) was calculated by measuring weight and height of the subject according to the equation weight in kilograms/height in meters squared. Active and sedentary lifestyle was based on whether the subject was doing a minimum of 30 min of brisk walking or aerobic exercise every day or not. Systolic (SBP) and diastolic blood pressure (DBP) were measured by taking an average of the three blood pressure readings using a sphygmomanometer, taken after 3 min interval each with the subject in resting position. Total cholesterol (TC), high density lipoprotein (HDL), low density lipoprotein (LDL) and triglycerides (TG) were tested using commercially available assay kits (Erba Mannheim, London, UK) with a minimum detection ceiling of 0.1 mg/L. The inter- and intra-assay coefficients of variation were 0.032 and 0.040 respectively for the measurement of these lipid values. All the postmenopausal women were examined for the quality of sleep they were experiencing according to the method of the Pittsburgh Sleep Quality Index (PSQI). It entails 7 domains of sleep through 19 questions, and these are quality of the sleep, duration of the sleep, latency of the sleep, efficiency of habitual sleep, medication use for sleep, disturbances during sleep and sleep dysfunction during the past one month. The sum of the scores reveals the global score. A global score of less than or greater than 5 indicates good sleep or poor sleep respectively. A global score greater than 5 calculated via the PSQI has been reported to have 89.6 percent sensitivity and 86.5 percent specificity for distinguishing good sleep and bad sleep [11]. Plasma levels of C-reactive protein (CRP) and nitric oxide (NO) (ThermoFisher Scientific, Waltham, MA, USA) were tested with an enzyme linked immunosorbent assay (ELISA) using a microplate reader (Biotek Instruments Inc., Winooski, VT, USA). Diagnostic sensitivities of the kits were 9.38 pg/mL and 1.75 pg/mL, respectively. Inter- and intra-assay coefficients of variation for CRP and NO were 0.039 and 0.044, respectively.

### 2.4. Statistical Analysis

Data are shown as number, percentages or mean ± standard deviation. To analyse the differences between the groups, a Chi-square and Student’s *t*-test were used for categorical and continuous variables, respectively. Univariable linear regression analysis was done to investigate the impact of risk variables. Those variables which showed significant relationships (*p* < 0.1) in univariable testing were further analysed via backward stepwise multivariable logistic regression analysis. *p* < 0.05 was considered to show significant associations, and for multiple comparisons Bonferroni’s correction was applied, wherever required.

## 3. Results

The present study involved 672 postmenopausal women, out of which 205 (30.50%) were osteoporotic, 297 (44.20%) were osteopenic and 170 (25.30%) had normal bone mass (Table 1). The mean ages of osteoporotic, osteopenic and control subjects were 68.5 ± 8.0, 69.7 ± 8.40 and 69.2 ± 8.1, respectively. The mean YSM was observed to be 12.8 ± 4.0 in osteoporotic participants, followed by 12.9 ± 5.7 in osteopenic participants, and control subjects were 13.7 ± 5.9 YSM. A total of 9.76% of osteoporotic and 11.45% of osteopenic subjects were observed to be smokers and 16.59% of osteoporotic and 11.11% of osteopenic subjects were alcohol drinkers. All the values of lipid profiles except HDL were observed to be the highest in osteoporotic subjects followed by osteopenic subjects and then in control subjects.

### Risk Factors Influencing Osteoporosis and Osteopenia

Univariable regression analysis by employing all the probable risk factors for osteoporosis and osteopenia (Table 2) revealed that higher body mass index (>30 Kg/m^−2^) is a protective factor against the risk of osteoporosis (OR 0.51 95%CI: 0.28–0.81, *p* = 0.004) and osteopenia (OR 0.58 95%CI: 0.32–0.92, *p* < 0.001). Age was observed to be insignificant between both groups (*p* > 0.05). Women having YSM >10 years had 1.84 and 1.96 odds of a higher risk of osteoporosis (OR 1.84 95%CI: 1.04–3.73, *p* = 0.026) and osteopenia (OR 1.96 95%CI: 1.44–3.09, *p* = 0.002), respectively. Similarly, higher SBP (>120 mmHg) and higher TG levels (>150 mg/dl) were observed to impact the risk of osteoporosis (OR 1.95 95%CI: 1.08–2.74, *p* = 0.009 & OR 2.72 95%CI: 1.93–3.93, respectively) and osteopenia (OR 1.93 95%CI: 1.22–2.86, *p* < 0.008 & OR 2.62 95%CI 1.78–3.91, *p* < 0.001, respectively). It is well known that lower BMD is a hallmark for osteoporosis and osteopenia, which has been endorsed by this study, as well as the fact that lesser values of BMD at the femoral neck (<0.7 g/cm^2^) and lumbar spine (<0.8 g/cm^2^) influence the risk of both osteoporosis and osteopenia (*p* < 0.05). Higher CRP levels (>3 mg/L) emerged to be the most significant univariable marker, which tripled the risk of osteoporosis (OR 3.28 95%CI: 2.65–4.22, *p* < 0.001) and osteopenia (OR 2.13 95%CI: 1.51–2.84, *p* = 0.005). Multiple backward stepwise regression analysis revealed that YSM >10 years was an independent predictor for osteopenia (OR 1.27 95%CI: 1.29–2.55, *p* = 0.033) but not for osteoporosis (*p* > 0.05). All risk variables which were significantly associated in the univariable analysis (except YSM) retained their significance in the multivariable model, influencing the risk of osteoporosis and osteopenia.

Factors which emerged to be independent predictors for the risk of osteoporosis were higher SBP levels (OR 1.23 95%CI: 1.22–3.11, *p* = 0.026), higher TG levels (OR 2.00 95%CI: 1.21–3.10, *p* = 0.005), poor sleep (OR 1.89 95%CI: 1.91–2.47, *p* = 0.014) and lesser BMD at femoral neck (OR 1.32 95%CI: 1.09–2.65, *p* = 0.023) and at lumbar spine (OR 1.21 95%CI: 1.10–2.59, *p* = 0.028). Higher CRP levels (>3 mg/L) emerged to be the highest independent predictor, bearers of which had 2.77 odds of higher risk of osteoporosis (OR 2.77 95%CI: 2.18–3.56, *p* = 0.008) than those who had lower levels of CRP. Higher BMI emerged to be the protective factor (OR 0.43 95%CI: 0.26–0.68, *p* = 0.02) against the risk of osteoporosis. Similarly, SBP (>120 mmHg), TG (>150 mg/dl), poor sleep (>5 scores), higher CRP (>3 mg/L) and lower femoral neck BMD (<0.7 g/cm^2^) and BMD at lumbar spine (<0.8 g/cm^2^) were independent risk factors for the risk of osteopenia. Women having higher BMI (>30 kg·m^−2^) were observed to be at 38 percent lesser chance (OR 0.38 95%CI: 0.19–0.52, *p* = 0.004) of getting osteopenia.

## 4. Discussion

The present study has examined the prevalence and predictors for the risk of osteoporosis and osteopenia in 672 postmenopausal women of Punjab, India. This is the first study from this region of India, which has revealed that the prevalence rate of osteoporosis (30.50%) is approximately 14% lower than that of osteopenia (44.20%). Higher BMI (>30 kg·m^−2^) has been observed to be an independent protective factor against the risk of osteoporosis and osteopenia. This inference is corroborated by a systematic review and meta-analysis [12], however, others have shown contrasting results exhibiting that higher BMI is associated with osteoporosis risk and fragility fractures [13,14]. Interestingly, it has been proved that fracture risk in postmenopausal women increases because of adiposity (higher BMI) but not because of low BMD [15]. The profound analysis of these contrasting and indecisive findings regarding BMI vis-à-vis osteoporosis risk suggests two points: firstly, the relationship of BMI with the development of osteoporosis is a consequence rather than a cause, which does not influence individually but other factors interact, for instance, sedentary lifestyle. Secondly, maintaining a normal weight is a better proposition after menopause. Therefore, modifying BMI with exercise may help in mitigating the risk of postmenopausal osteoporosis.

Few reports have shown a strong correlation of higher lipid profiles, especially triglycerides, with impaired bone mass, attenuated bone mineral metabolism and increased fragility fractures [16,17]. Triglycerides are produced in the liver, which are carried by very low-density lipoprotein (VLDL). These uncleared TGs because of impaired LDL mediated transport help in the supplementation of reaction oxygen species (ROS) causing oxidation of lipids. These oxidized lipids inhibit osteoblast differentiation both in arterial walls and in bone, hence, inhibiting bone mineral formation [18]. In the present study, it has been observed that those women who have higher TG levels (>150 mg/dl) have double the risk of osteoporosis (OR 2.00 95%CI: 1.21–3.10, *p* = 0.005) and 1.63 odds of higher risk of osteopenia (OR 1.63 95%CI: 1.42–2.51, *p* = 0.038). It has been observed that lipid lowering drugs, especially statins (HMG-CoA reductase inhibitor), increase bone mineralization and significantly reduce the risk of fractures [19,20]. As lipids can be modified either by regulating dietary intake or via pharmacological interventions, the risk of osteoporosis owing to higher lipids can be curtailed to some extent.

It has been clarified via a meta-analysis that hypertension is associated with bone loss and higher risk of fractures [21], which has been exposed in the present study also whereby higher SBP (>120 mmHg) is observed to be independently associated with risk of osteoporosis and osteopenia. Hypertension has been implicated in severe loss of bone minerals including calcium and its metabolism, resulting in accelerated bone resorption [22]. It is now known that anti-hypertensive drugs such as thiazides reduce the risk of fractures by supplementing bone minerals to the bone. Moreover, the activated osteoclasts due to the angiotensin II (Ang II)-induced receptor activator of the NF-κB ligand (RANKL) pathway can be suppressed with AngII inhibitors, which reduce the risk of osteoporotic fractures manifold [23]. In this regard, it is recommended that the ill effects of hypertension on bone can be managed using an anti-hypertensive drug regimen.

Sound sleep involving adequate time is important for bone health, whereas poor sleep may affect skeletal health by causing deformity and reducing the ability of bone to heal from fragility fractures [24]. Apropos to this, the present study has found that postmenopausal women suffering from poor sleep (≥5 PSQI scores) are approximately doubly vulnerable to the risk of osteoporosis and osteopenia. This is endorsed by the inference of the study that individuals who have 8 h of sound sleep have lower chances of osteoporosis than those who sleep for 6 h [25]. Although advancing age is significantly related with disturbed sleep, sleep apnea and sleep disorders, its impact on health can be managed using a variety of remedies such as taking a hot shower before going to bed, doing meditation, listening to music and going for a walk after dinner. However, in severe cases of sleep apnea and sleep disorders, pharmacological management is required. Therefore, sleep is considered to be a partially modifiable risk factor which can be corrected with targeted and personalized therapy.

In the clinical chapters, it is suggested that chronic inflammation reduces BMD and higher levels of the inflammatory marker CRP is inversely associated with BMD and, hence, is directly associated with the risk of osteoporosis and fractures [26]. This is endorsed by the present study, where higher CRP levels have emerged to be the strongest risk factor for osteoporosis and osteopenia. Plasma CRP is a clinical entity that is strongly associated with modifiable factors such as exercise. Comprising several cross sectional and longitudinal studies, a systematic review has exhibited that exercise significantly reduces CRP levels [27]. Another study has shown that higher CRP levels are significantly correlated with lower BMD in healthy pre- and postmenopausal women [28]. All these studies suggest that lowering CRP via exercise or anti-inflammatory medicines can be useful in alleviating the risk of bone mineral loss and fractures.

## 5. Conclusions

A higher BMI protects against the risk of osteoporosis and osteopenia, whereas a higher SBP and higher levels of plasma CRP and TG along with poor sleep independently enhance the risk of osteoporosis and osteopenia in postmenopausal women of Punjab, India. The higher prevalence of osteoporosis (30.50%) and osteopenia (44.20%) in postmenopausal women of this region is disturbing and suggests that every woman attaining menopause should be tested and given preliminary awareness of osteoporosis, so that better health care of postmenopausal women can be assured.

### Strengths and Limitations of the Study

This is the first study from this region which examined not only the prevalence of osteoporosis and osteopenia but also exposed independent risk factors for these conditions. Nonetheless, some limitations in this study may have blurred the findings, for instance, information on some risk factors like economic status, occupation and social behaviour could not be collected, which may have infused residual confounding. More studies covering all these aspects may clear the overall picture of osteoporosis and osteopenia risk in this region of India.

## Figures and Tables

**Table 1:** General characteristics of the study participants.

Variables	Women with Normal Bone Mass (*n* = 170)	Women with Osteoporosis (*n* = 205)	Women with Osteopenia (*n* = 297)	† *p* Value	‡ *p* Value
Age (years)	69.2 ± 8.1	68.5 ± 8.0	69.7 ± 8.4	0.40	0.53
YSM (years)	13.7 ± 5.9	12.8 ± 4.0	12.9 ± 5.7	0.08	0.15
BMI (kg·m^−2^)	24.6 ± 1.9	23.5 ± 1.3	23.9 ± 1.6	**<0.001**	**<0.001**
SBP (mmHg)	124.15 ± 12.45	128.43 ± 13.60	127.40 ± 12.10	**0.002**	**0.006**
DBP (mmHg)	96.8 ± 11.25	99.07 ± 14.34	97.72 ± 13.32	0.09	0.45
TC (mg/dl)	224.89 ± 21.04	227.67 ± 21.05	225. 45 ± 20.36	0.20	0.78
LDL (mg/dl)	195.10 ± 19.74	197.99 ± 14.30	196.86 ± 9.30	0.10	0.19
HDL (mg/dl)	49.26 ± 4.87	48.99 ± 3.69	49.18 ± 3.98	0.54	0.85
TG (mg/dl)	133.46 ± 47.32	170.43 ± 57.99	163.79 ± 55.87	**<0.001**	**<0.001**
Non-Smokers	139 (81.77)	169 (82.44)	241 (81.14)	0.89	0.97
Smokers	21 (12.35)	20 (9.76)	34 (11.45)		
Ex-Smokers	10 (5.88)	16 (7.80)	22 (7.41)		
Non-Drinkers	143 (84.12)	155 (75.61)	245 (82.49)	0.26	0.95
Drinkers	15 (8.82)	34 (16.59)	33 (11.11)		
Ex-drinkers	12 (7.06)	16 (7.80)	19 (6.40)		
Family history, Yes	69 (40.59)	124 (60.49)	162 (54.55)	**<0.001**	**0.004**
Family history, No	101 (59.41)	81 (39.51)	135 (45.45)		
Physically Active	106 (62.35)	89 (43.41)	139 (46.80)	**<0.001**	**0.001**
Sedentary	64 (37.65)	116 (56.59)	158 (53.20)		
Good sleep (<5 score)	105 (61.76)	87 (42.44)	141 (47.47)	**<0.001**	**0.003**
Poor sleep (≥5 score)	65 (38.24)	118 (57.56)	156 (52.53)		
BMD_FN (g/cm^2^)	0.94 ± 0.09	0.84 ± 0.19	0.88 ± 0.15	**<0.001**	**<0.001**
BMD_LS (g/cm^2^)	0.90 ± 0.05	0.73 ± 0.18	0.80 ± 0.23	**<0.001**	**<0.001**
CRP (mg/L)	5.88 ± 7.6	14.03 ± 7.2	11.99 ± 7.0	**<0.001**	**<0.001**
NO (μmol/L)	9.1 ± 5.8	8.3 ± 5.05	9.0 ± 5.5	0.15	0.85

Values are given as both number and percentages or mean ± standard deviation. † *p* value: control subjects versus osteoporosis subjects. ‡ *p* value: control subjects versus osteopenic subjects. YSM: years since menopause, BMI: body mass index, SBP: systolic blood pressure, DBP: diastolic blood pressure, TC: total cholesterol, LDL: low density lipoprotein, HDL: high density lipoprotein, TG: triglycerides, BMD_FN: bone mineral density at femoral neck, BMD_LS: bone mineral density at lumbar spine, CRP: C-reactive protein, NO: nitric oxide. Significant values are shown in bold face.

**Table 2:** Independent predictors for the risk of osteoporosis and osteopenia.

Variables	Input Variables	Univariable Analysis		Multivariable Analysis	
		OR (95%CI)	*p* Values	OR (95%CI)	*p* Values
Age (years)	≤60 vs. >60	1.22 (0.66–2.79) ^Ost^ 1.34 (0.82–3.00) ^Osp^	0.195 0.234	------	----
BMI (kg·m^−2^)	≤30 vs. >30	0.51 (0.28–0.81) ^Ost^ 0.58 (0.32–0.92) ^Osp^	**0.004** **<0.001**	0.43 (0.26–0.68) ^Ost^ 0.38 (0.19–0.52) ^Osp^	**0.020** **0.004**
YSM (years)	≤10 vs. >10	1.84 (1.04–3.73) ^Ost^ 1.96 (1.44–3.09) ^Osp^	**0.026** **0.002**	1.62 (0.96–2.77) ^Ost^ 1.27 (1.29–2.55) ^Osp^	0.070 **0.033**
DBP (mmHg)	≤80 vs. 80	1.64 (0.88–2.82) ^Ost^ 1.49 (0.82–2.03) ^Osp^	0.192 0.233	---------	---
SBP (mmHg)	≤120 vs. >120	1.95 (1.08–2.74) ^Ost^ 1.93 (1.22–2.86) ^Osp^	**0.009** **0.008**	1.23 (1.22–3.11) ^Ost^ 1.10 (1.08–2.49) ^Osp^	**0.026** **0.043**
TC (mg/dl)	≤200 vs. >200	1.40 (0.89–3.19) ^Ost^ 1.30 (0.78–3.10) ^Osp^	0.201 0.219	---------	---
LDL (mg/dl)	≤100 vs. >100	2.05 (0.59–2.10) ^Ost^ 2.53 (0.88–3.12) ^Osp^	0.189 0.284	---------	---
HDL (mg/dl)	≥40 vs. <40	1.52 (0.62–2.11) ^Ost^ 1.60 (0.74–2.67) ^Osp^	0.144 0.221	---------	---
TG (mg/dl)	≤150 vs. >150	2.72 (1.93–3.93) ^Ost^ 2.62 (1.78–3.91) ^Osp^	**<0.001** **<0.001**	2.00 (1.21–3.10) ^Ost^ 1.63 (1.42–2.51) ^Osp^	**0.005** **0.038**
Sleep (scores)	≤5 vs. > 5	2.53 (2.10–3.71) ^Ost^ 3.11 (2.75–4.61) ^Osp^	**<0.001** **<0.001**	1.89 (1.91–2.47) ^Ost^ 2.28 (1.76–3.47) ^Osp^	**0.014** **0.027**
CRP (mg/L)	≤3 vs. > 3	3.28 (2.65–4.22) ^Ost^ 2.13 (1.51–2.84) ^Osp^	**<0.001** **0.005**	2.77 (2.18–3.56) ^Ost^ 1.93 (1.03–2.18) ^Osp^	**0.008** **0.042**
NO (μmol/L)	≤11vs. >11	1.62 (0.78–1.29) ^Ost^ 1.68 (0.82–1.63) ^Osp^	0.382 0.349	----------	---
BMD_FN (g/cm^2^)	≥0.7 vs. <0.7	1.62 (1.32–3.78) ^Ost^ 1.43 (1.11–2.88) ^Osp^	**0.007** **0.007**	1.32 (1.09–2.65) ^Ost^ 1.12 (1.03–2.55) ^Osp^	**0.023** **0.034**
BMD_LS (g/cm^2^)	≥0.8 vs. <0.8	1.52 (1.29–3.28) ^Ost^ 1.22 (1.01–2.63) ^Osp^	**0.005** **0.023**	1.21 (1.10–2.59) ^Ost^ 1.13 (1.01–2.19) ^Osp^	**0.028** **0.041**

BMI: body mass index, YSM: years since menopause, DBP: diastolic blood pressure, SBP: systolic blood pressure, TC: total cholesterol, LDL: low density lipoprotein, HDL: high density lipoprotein, TG: triglycerides, CRP: C-reactive protein, NO: nitric oxide. BMD_FN: bone mineral density at femoral neck, BMD_LS: bone mineral density at lumbar spine, CI: confidence intervals. ^Ost^: osteoporosis, ^Osp^: osteopenia. Significant values are shown in bold face.

## Data Availability

Data shown in this paper are available on request from the corresponding author. The data are not publicly available due to ethical issues.
